# Comprehensive Analysis of lncRNAs Related to the Prognosis of Esophageal Cancer Based on ceRNA Network and Cox Regression Model

**DOI:** 10.1155/2020/3075729

**Published:** 2020-12-10

**Authors:** Chao Li, Wu Yao, Congcong Zhao, Guo Yang, Jingjing Wei, Yuanmeng Qi, Ruoxuan Huang, Qiuyan Zhao, Changfu Hao

**Affiliations:** College of Public Health, Zhengzhou University, 450000, China

## Abstract

**Background:**

Esophageal cancer is one of the most deadly malignant tumors. Among the common malignant tumors in the world, esophageal cancer is ranked seventh, which has a high mortality rate. Long noncoding RNAs (lncRNAs) play an important role in the occurrence and development of various tumors. lncRNAs can competitively bind microRNAs (miRNAs) with mRNA, which can regulate the expression level of the encoded gene at the posttranscriptional level. This regulatory mechanism is called the competitive endogenous RNA (ceRNA) hypothesis, and ceRNA has important research value in tumor-related research. However, the regulation of lncRNAs is less studied in the study of esophageal cancer.

**Methods:**

The Cancer Genome Atlas (TCGA) database was used to download transcriptome profiling data of esophageal cancer. Gene expression quantification data contains 160 cancer samples and 11 normal samples. These data were used to identify differentially expressed lncRNAs and mRNAs. miRNA expression data includes 185 cancer samples and 13 normal samples. The differentially expressed RNAs were identified using the edgeR package in R software. Then, the miRcode database was used to predict miRNAs that bind to lncRNAs. MiRTarBase, miRDB, and TargetScan databases were used to predict the target genes of miRNAs. Cytoscape software was used to draw ceRNA network. Gene Ontology (GO) and Kyoto Encyclopedia of Genes and Genomes (KEGG) analyses were performed using DAVID 6.8. Finally, multifactor cox regression was used to screen lncRNAs related to prognosis.

**Results:**

We have screened 1331 DElncRNAs, 3193 DEmRNAs, and 162 DEmiRNAs. Among them, the ceRNA network contains 111 lncRNAs, 11 miRNAs, and 63 DEmRNAs. Finally, we established a prediction model containing three lncRNAs through multifactor Cox regression analysis.

**Conclusions:**

Our research screened out three independent prognostic lncRNAs from the ceRNA network and constructed a risk assessment model. This is helpful to understand the regulatory role of lncRNAs in esophageal cancer.

## 1. Introduction

Esophageal cancer (EC) is one of the most deadly malignant tumors. Among the common malignant tumors in the world, EC is ranked seventh, which has a high mortality rate [1]. There are two main subtypes of esophageal cancer: esophageal squamous cell carcinoma (ESCC) is mainly distributed in Asia, Africa, and South America; esophageal adenocarcinoma (EAC) is mainly distributed in North America and Europe [2, 3]. Nowadays, surgical resection can improve the quality of life of patients with EC and prolong the survival time of patients, but the risk of surgical treatment is high. In addition, the use of surgical aids such as chemotherapy or chemoradiation to treat esophageal cancer has improved the prognosis of patients with advanced cancer, but the five-year survival rate of esophageal cancer is only 15%-25% [4–6]. Therefore, there is an urgent need to find molecular biomarkers for EC, which can help improve the prognosis and treatment of patients with EC.

Long noncoding RNA (lncRNA) is defined as RNA transcripts with more than 200 nt and no coding ability [7]. There is increasing evidence that lncRNAs can regulate tumor genesis, including tumor cell proliferation, metastasis, differentiation, apoptosis, and metabolism [8–10]. Among them, MALAT1, AFAP1-AS1, HOTAIR, TUG1, and MEG3 have been shown to be dysregulated in EC and they can regulate the occurrence and development of EC [11–16]. In addition, related studies have shown that lncRNAs may become prognostic markers for EC [17].

lncRNAs can regulate tumor genesis in many ways. When located in the nucleus, they are mainly involved in the process of transcription and epigenetics. When located in the cytoplasm, they participate in posttranscriptional regulation mainly by forming specific protein complexes or as ceRNA [18]. In 2011, Salmena et al. proposed the hypothesis of competitive endogenous RNA [19]. With the deepening of research related to lncRNA, the research of competitive endogenous RNA hypothesis is also increasing, which has become a hotspot in disease research.

In our study, the expression data of lncRNAs, miRNAs, and mRNAs related to EC samples and normal samples came from TCGA database. The differentially expressed RNAs were selected. Subsequently, Gene Ontology (GO) and Kyoto Encyclopedia of Genes and Genomes (KEGG) analyses were used to reveal the potential biological mechanisms of differentially expressed mRNAs. Then, we successfully constructed an lncRNA-related ceRNA network in EC after differential expression analysis and database comparison. Finally, univariate and multivariate COX regressions were used to find lncRNAs related to prognosis. Our research has found lncRNAs related to the prognosis of EC. These lncRNAs may become markers of EC prognosis.

## 2. Materials and Methods

### 2.1. Data Collection and Preprocessing

RNA-seq data, microRNA data, and the clinical data of EC were downloaded from The Cancer Genome Atlas (TCGA) database (https://cancergenome.nih.gov/). RNA-seq data contains 160 cancer samples and 11 normal samples. These data were used to identify differentially expressed lncRNAs and mRNAs. MicroRNA data includes 185 cancer samples and 13 normal samples. Perl (version 5.28.1; https://www.perl.org/) was used for data processing. Ensemble database (Ensemble release 99; http://asia.ensembl.org/index.html) was used for gene annotations and identification of lncRNAs and mRNAs. RNAs that have not been annotated by the database were excluded. Our research was conducted in accordance with TCGA publication guidelines. Therefore, the approval from the local ethics committee was not required.

### 2.2. Identification of Differentially Expressed RNAs

RNAs with no expression or average count ≤ 1 will be filtered. The differentially expressed RNAs were identified using the edgeR package in R (version3.5.1) [20]. ∣log2 fold change (FC) | >1 and false discovery rate (FDR) < 0.05 were the screening condition for differential RNAs. In addition, the heat maps and volcano maps of differentially expressed RNAs were drawn by the gplots and heat map packages in the R software.

### 2.3. Gene Ontology and KEGG Enrichment Analysis

DAVID 6.8 (https://david.ncifcrf.gov) was used for Gene Ontology (GO) enrichment analysis and Kyoto Gene and Genome Encyclopedia (KEGG) signal pathway analysis based on DEmRNAs. GO enrichment analysis can classify and annotate genes through three aspects: biological path (BP), cellular component (CC), and molecular function (MF). The KEGG signaling pathway was used to find important signaling pathways.

### 2.4. Construction of ceRNA Network

**Figure 1:**
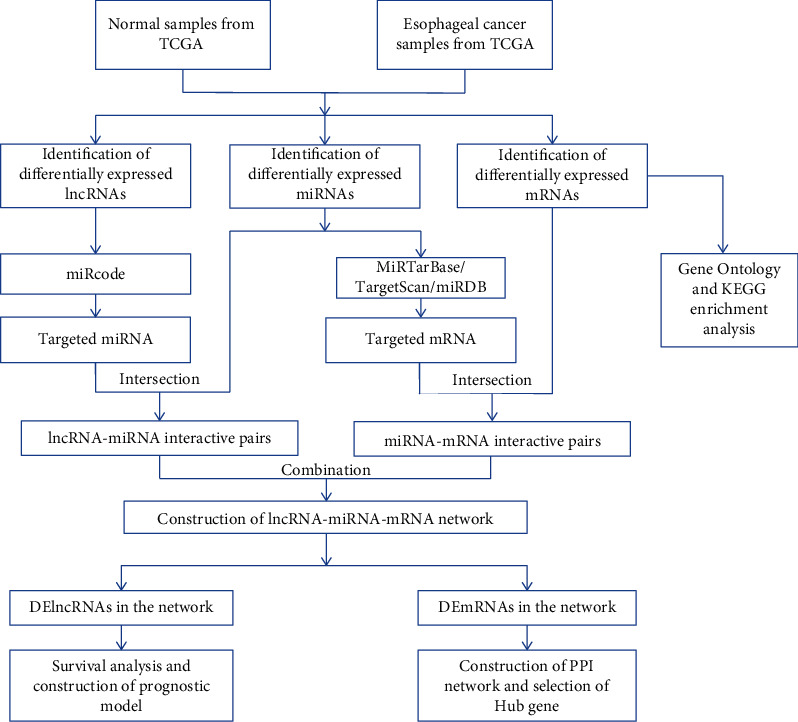
ceRNA network construction flowchart.

The miRcode database (http://www.mircode.org/) was used to predict miRNAs that bind to lncRNAs. MiRTarBase (http://mirtarbase.cuhk.edu.cn/), miRDB (http://www.mirdb.org/), and TargetScan (http://www.targetscan.org/) databases were used to predict the target genes of miRNAs [21–23]. The mRNAs obtained from the overlapping parts of the three data sets and intersected with DEmRNAs were considered to be mRNAs bound to miRNAs. Then, we constructed a coexpression network of differently expressed RNAs based on the DElncRNA-DEmiRNA and DEmiRNA-DEmRNA interactions. Cytoscape (version 3.7.1) was used for network visualization. The flow chart of ceRNA network construction was displayed (see Figure 1).

### 2.5. Construction of PPI Network and Selection of Hub Gene

The STRING (search tool for the retrieval of interacting genes) (https://string-db.org) database was used to construct a protein interaction network for differential genes in the ceRNA network, and the medium credibility (interaction score > 0.4) was the screening criterion [24]. Cytoscape (version 3.7.1) was used for PPI network visualization. The maximal clique centrality (MCC) method from the cytoHubba app in Cytoscape was used to screen for genes with higher scores, which were considered key genes [25, 26].

### 2.6. Survival Analysis and Prognostic Model

The “survival” package in R software was used for survival analysis of DElncRNAs in the ceRNA network. The Kaplan-Meier method was used to draw survival curves, and the log-rank test was used to compare the differences between the two groups. *p* < 0.05 was considered statistically significant. Subsequently, univariate COX regression was used to evaluate the association between DElncRNAs in the ceRNA network and the overall survival rate of EC, and *p* < 0.05 was considered to be significant. Then, DElncRNAs from univariate cox regression and LR analysis were used to construct a multivariate Cox proportional hazards regression model. The multivariate Cox regression model was used to construct a prognostic model of DElncRNAs in the ceRNA network. The stepwise regression method was used to include and exclude variables. This method is based on Akaike Information Criterion (AIC). AIC is a standard used to measure the goodness of a statistical model. It is generally considered that the model with the smaller AIC value is the optimal model. The model expression formula is as follows:

(1) $$\begin{array}{c} \text{Risk score}=CoencRNA1*ExplncRNA1+CoenlncRNA2*ExplncRNA2+\cdots CoencRNAn*ExplncRNAn. \end{array}$$

“Coe” represents the regression coefficient of the multiple COX regression model, and “Exp” represents the expression level of lncRNAs.

According to the median value of risk score, EC patients were divided into high- and low-risk groups. Kaplan-Meier analysis was used to compare the overall survival rates of the two groups. The “timeROC” package was used to plot the time-dependent receiver operating characteristic (ROC) curve, which can evaluate the value of the model prediction.

## 3. Results

### 3.1. Identification of the DElncRNAs, DEmiRNAs, and DEmRNAs

**Figure 2:**
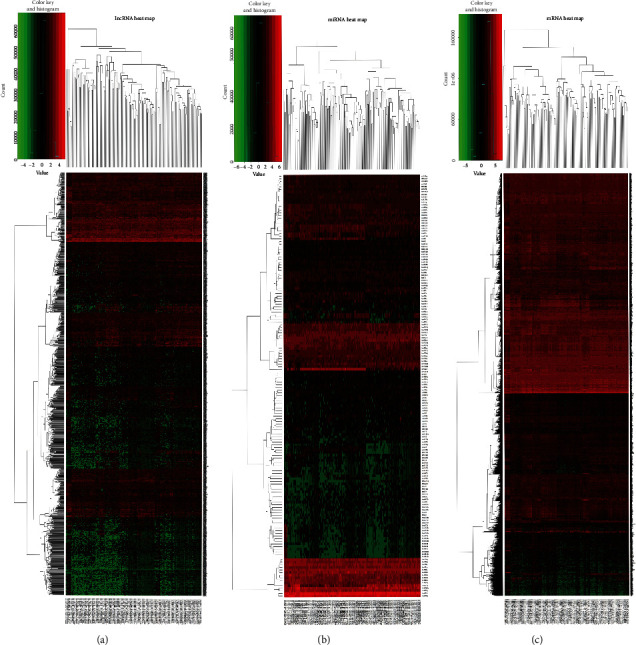
Heat maps of differential expression of lncRNAs, miRNAs, and mRNAs: (a) heat map of differential expression of lncRNAs; (b) heat map of differential expression of miRNAs; (c) heat map of differential expression of mRNAs.

**Figure 3:**
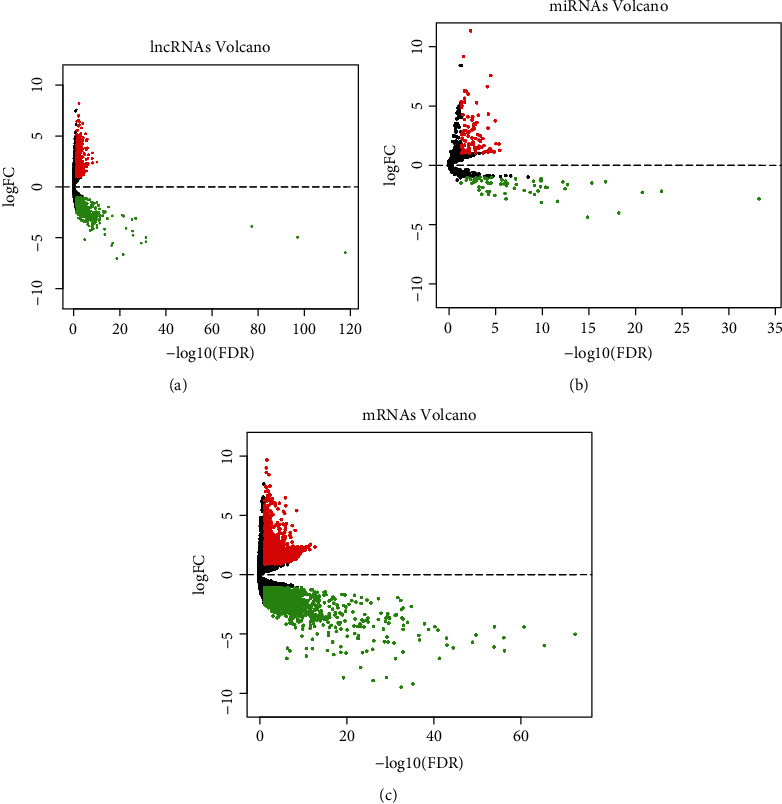
Volcano maps of differential expression of lncRNAs, miRNAs, and mRNAs: (a) volcano map of differential expression of lncRNAs; (b) volcano map of differential expression of miRNAs; (c) volcano map of differential expression of mRNAs. Red represents upregulated RNAs, and green represents downregulated RNAs.

The differentially expressed RNAs were identified by using the edgeR package in R software. ∣log2 fold change (FC) | >1 and false discovery rate (FDR) < 0.05 were the screening condition for differential RNAs. 1331 DElncRNAs (648 downregulated and 683 upregulated) and 3193 DEmRNAs (1753 downregulated and 1440 upregulated) were screened out of 160 EC samples and 11 normal samples. 162 DEmiRNAs (64 downregulated and 98 upregulated) were screened out of 185 cancer samples and 13 normal samples. The heat map of differential RNAs is displayed (see Figure 2). The volcano diagram of differential RNAs is displayed (see Figure 3). The results indicated that these differential RNAs might play a role in the development of EC.

### 3.2. Functional Analysis of DEmRNAs

**Figure 4:**
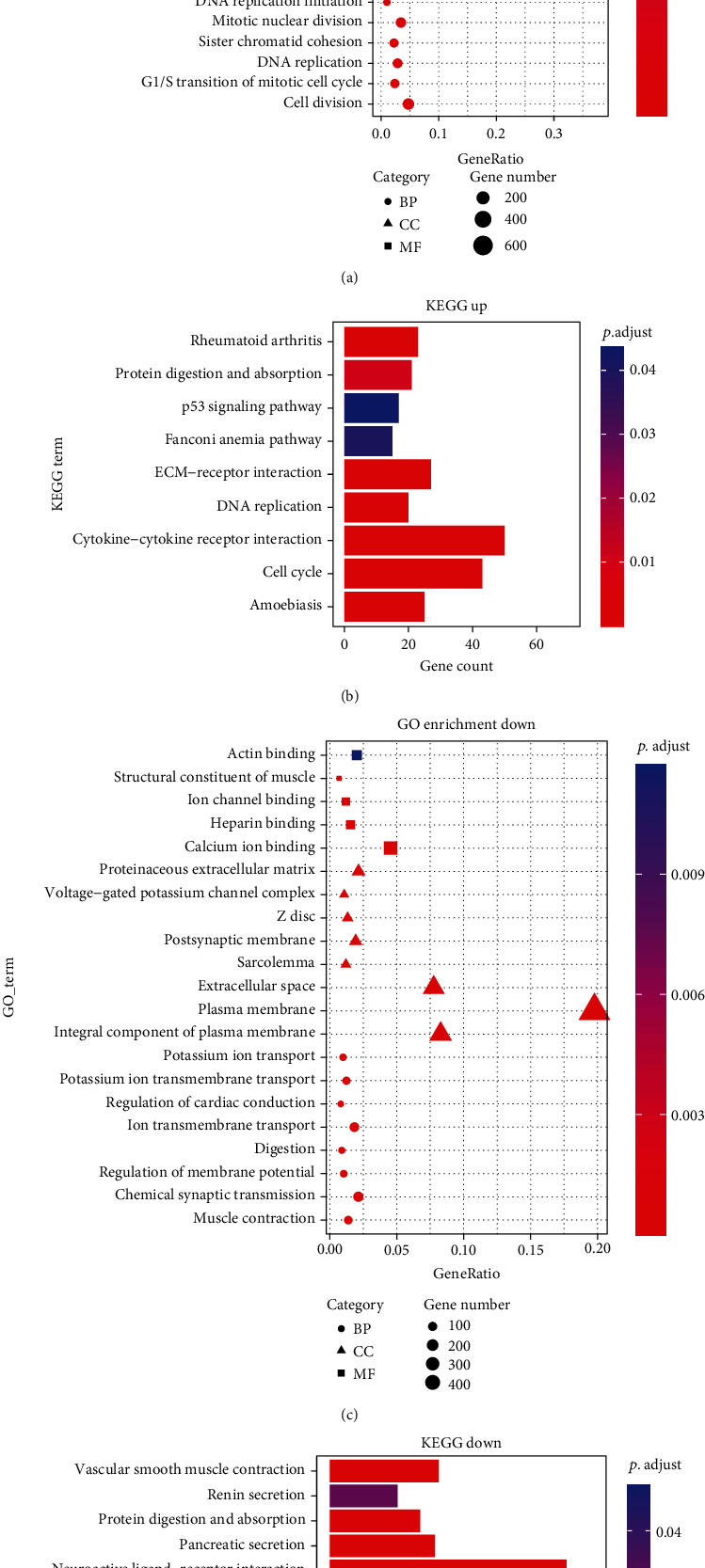
The gene ontology (GO) enrichment analysis of DEmRNAs and the bubble chart and bar chart of the Kyoto Encyclopedia of Gene and Genome (KEGG) signaling pathway analysis: (a) GO enrichment analysis of upregulated DEmRNAs; (b) analysis of the KEGG signaling pathway of upregulated DEmRNAs; (c) GO enrichment analysis of downregulated DEmRNAs; (d) analysis of KEGG signaling pathway of downregulated DEmRNAs.

DAVID 6.8 (https://david.ncifcrf.gov) was used for Gene Ontology (GO) enrichment analysis and Kyoto Gene and Genome Encyclopedia (KEGG) signal pathway analysis based on DEmRNAs. GO enrichment analysis can classify and annotate genes through three aspects: biological path (BP), cellular component (CC), and molecular function (MF). Upregulated mRNAs were classified as 47 biological process (BP) terms, 20 cellular component (CC) terms, and 8 molecular function (MF) terms using GO enrichment analysis. Downregulated mRNAs were classified as 10 biological process (BP) terms, 12 cellular component (CC) terms, and 5 molecular function (MF) terms using GO enrichment analysis. The results of partial GO enrichment are displayed (see Figures 4(a) and 4(c)). Subsequently, KEGG signaling pathway analysis showed that upregulated mRNAs were significantly enriched in 9 signaling pathways, and downregulated mRNAs were significantly enriched in 12 signaling pathways (see Figures 4(b) and 4(d)). The upregulated mRNAs were mainly involved in cell cycle, DNA replication, cytokine-cytokine receptor interaction, and ECM-receptor interaction. Downregulated mRNAs were mainly involved in neuroactive ligand-receptor interaction, gastric acid secretion, calcium signaling pathway, and pancreatic secretion. These results can help us understand the key signaling pathways and biological processes in the development of EC.

### 3.3. Construction of ceRNA Network

**Figure 5:**
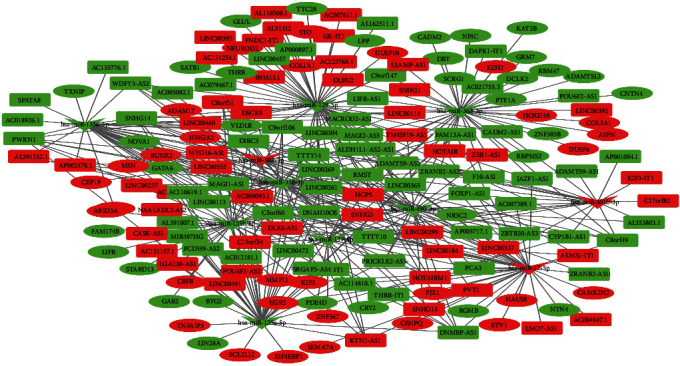
the ceRNA network of lncRNA-miRNA-mRNA in EC. Rectangles represent lncRNAs, V represents miRNAs, and ellipses represent mRNAs. The red nodes are upregulated RNAs, and the green nodes are downregulated RNAs.)

Bioinformatics tools were used to predict the interaction between DElncRNAs, DEmiRNAs, and DEmRNAs. The miRcode database (http://www.mircode.org/) was used to predict miRNAs that bind to lncRNAs. The Perl language was used to extract DElncRNAs and miRNAs combined with DElncRNAs in the miRcode database. Then, the DEmiRNAs and the miRNAs extracted from the miRcode database were intersected to obtain the miRNAs in the ceRNA network. The lncRNAs targeted by these miRNAs were the lncRNAs in the ceRNA. MiRTarBase, miRDB, and TargetScan databases were used to predict the target genes of miRNAs in the ceRNA network. After these target genes intersect with DEmRNAs, they were the mRNAs in the ceRNA network. In the end, 111 lncRNAs (62 downregulated and 49 upregulated), 11 miRNAs (8 downregulated and 3 upregulated), and 63 mRNAs (33 downregulated and 30 upregulated) were included in the ceRNA network. The lncRNAs, miRNAs, and mRNAs in the ceRNA network are displayed (see Table 1). Cytoscape was used for network visualization. EC-related ceRNA network is displayed (see Figure 5).

### 3.4. Construction of PPI Network and Selection of Hub Gene

**Figure 6:**
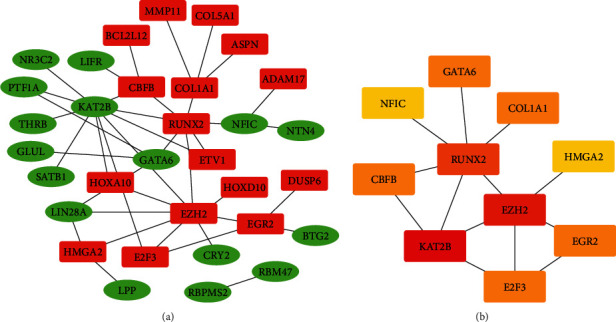
Identification of hub genes from the PPI network with the MCC method. (a) There are 32 genes in the PPI network. The red nodes are upregulated genes, and the green nodes are downregulated genes. (b) Top ten key genes screened by the MCC method; red was the higher score calculated by the MCC method, followed by orange.

The STRING (search tool for the retrieval of interacting genes) database (https://string-db.org) was used to construct a protein interaction network of differential genes. Medium credibility (interaction score > 0.4) was used as the screening criterion, loose links and outliers were removed, and the PPI network was drawn. Cytoscape was used for PPI network visualization. The PPI network is displayed (see Figure 6(a)). The maximal clique centrality (MCC) method from the cytoHubba app in Cytoscape was used to screen for genes with higher scores, which were considered key genes. The key genes are displayed (see Figure 6(b), Table 2). Our results indicated that KAT2B, EZH2, RUNX2, COL1A1, E2F3, CBFB, EGR2, GATA6, NFIC, and HMGA2 had important roles in the ceRNA network. These genes might be key genes in the development of EC.

### 3.5. Survival Analysis and Prognostic Model Construction

**Figure 7:**
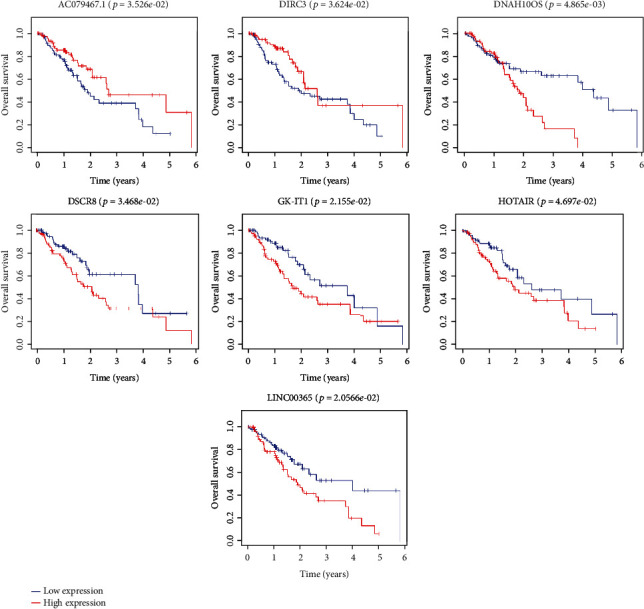
Kaplan-Meier survival curves of seven DElncRNAs associated with overall survival in EC.

**Figure 8:**
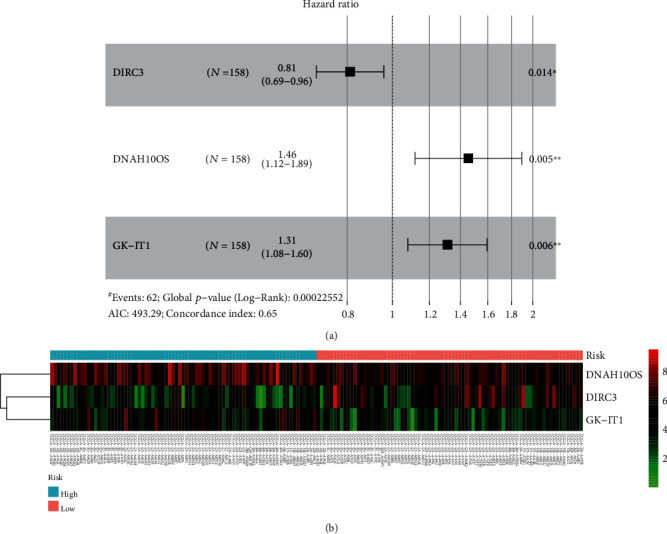
The prognostic model containing 3 lncRNAs was constructed by multiple Cox regression. (a) The prognostic model contains a forest plot of hazard ratios of three lncRNAs. (b) Heat map of the expression levels of the three lncRNAs included in the model in the high- and low-risk groups.

More and more evidences have shown that lncRNAs can predict the overall survival rate of cancer patients. We identified lncRNAs related to prognosis from related DElncRNAs in the ceRNA network. The Kaplan-Meier method was used to draw survival curves, and log-rank test was used to compare the differences between the two groups. Seven lncRNAs were considered to be related to the overall survival rate of EC (see Figure 7). AC079467.1 and DIRC3 were considered to be positively correlated with overall survival. DNAH10OS, DSCR8, GK-IT1, HOTAIR, and LINC00365 were considered to be negatively correlated with overall survival. Then, univariate cox regression analysis was used for further analysis. Our results showed that only three lncRNA univariate cox regression results were statistically significant in seven lncRNAs. Multivariate cox regression was used to construct a prognostic model related to lncRNAs. All three lncRNAs were included in the model. Among them, DIRC3 might be a protective factor for the prognosis of OS (HR < 1, see Figure 8(a)). DNAH10OS and GK-IT1 might be risk factors for the prognosis of OS (HR > 1, see Figure 8(a)). The model expression formula is as follows:

(2) $$\begin{array}{c} \text{Risk score}=-0.20910*{Exp}_{\text{DIRC}3}+0.37509*{Exp}_{\text{DNAH}10\text{OS}}+0.27170*{Exp}_{\text{GK}­\text{IT}1}. \end{array}$$

According to the median value of risk score, EC patients were divided into high- and low-risk groups. The heat map of the expression profiles of the three lncRNAs in EC patients is displayed (see Figure 8(b)).

**Figure 9:**
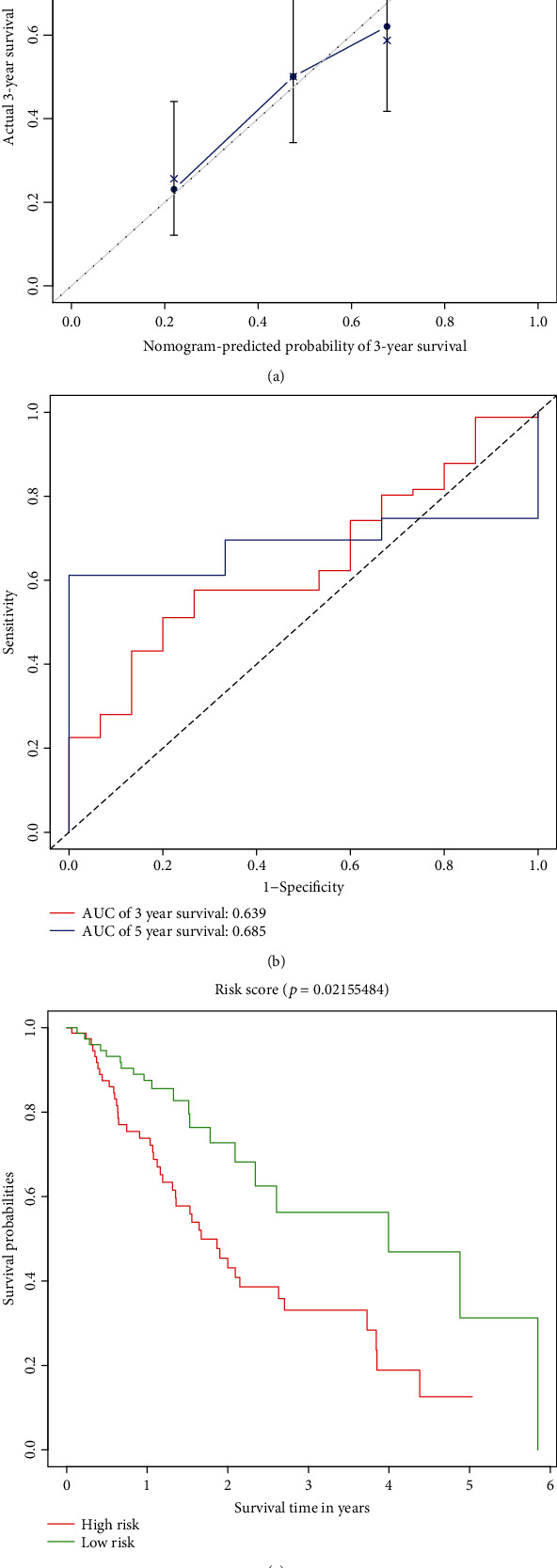
Evaluation of model prognostic ability. (a) The calibration curves of the 3-year overall survival rate; the abscissa was the predicted mortality of the model, and the ordinate was the actual mortality. (b) The receiver operating characteristic (ROC) curve of the three-year survival rate and the five-year survival rate predicted by the model. (c) Kaplan-Meier plots of overall survival for patients with a high- or low-risk score of the EC patients.

Among them, there were 79 samples of EC patients in the high-risk group and the low-risk group respectively. In order to reflect the predictive performance of the model, K-M analysis was used to compare the overall survival rate of the high-risk group and the low-risk group. The results showed that the overall survival rate between the two groups was statistically different (see Figure 9(c), *p* < 0.05). The calibration curve was used to test the consistency between the model's predicted mortality rate and the actual mortality rate. The calibration curves of the 3-year overall survival rate (see Figure 9(a)) showed that there was good agreement between the predicted mortality rate and the actual mortality rate. In addition, our model was used to predict the 3-year survival rate and 5-year survival rate of patients with EC. The time-dependent receiver operating characteristic (ROC) curves are displayed (see Figure 9(b)). The area under the curve for 3-year survival rate was 0.639, and the area under the curve for 5-year survival rate was 0.685. These results indicated that the PI of lncRNAs showed a good prognostic ability, which suggested that the prognostic model constructed by the three lncRNAs may be a prognostic factor for EC.

## 4. Discussion

Esophageal cancer (EC) is one of the most deadly malignant tumors. The five-year survival rate of EC is only 15%-25% [4–6]. Therefore, there is an urgent need to find molecular biomarkers for EC, which can help improve the prognosis and treatment of patients with EC. Long noncoding RNA has been confirmed to be associated with poor prognosis in lung cancer [27, 28], gastric cancer [29, 30], liver cancer [31], and other cancers [32].

lncRNAs can regulate the occurrence of diseases in a variety of ways. Existing studies have shown that lncRNAs can interact with epigenetics to regulate disease processes in the nucleus; these interactions include the interaction between lncRNAs and DNA methylation [33]. lncRNAs can also interact with transcription factors to play an important role at the transcription level [34]. lncRNAs can directly bind to mRNA to increase its stability or make it degrade, inhibit, or promote its translation in the cytoplasm [18]. However, more research has focused on lncRNAs as a member of the ceRNA network to play its regulatory role, which has even become the research focus of lncRNAs in disease-related roles.

More and more evidence has shown that lncRNAs participate in the regulation of EC through the ceRNA network mechanism. lncRNA MALAT1 can regulate miR-101 and miR-207 to affect the proliferation, invasion, and metastasis of ESCC cells [11]. SNHG16, which is upregulated in ESCC tissues and cell lines, can bind miR-140-5p to regulate the expression of ZEB1 [35]. The expression of lncRNA HAND2-AS1 is downregulated in tumor tissue. The overexpression of lncRNA HAND2-AS1 may inhibit the proliferation, migration, and invasion of cancer cells in ESCC by downregulating miRNA-21 [36]. These studies indicated that lncRNAs can play a role through the ceRNA network in EC, and lncRNAs are closely related to the occurrence of tumors. Therefore, our study screened differentially expressed lncRNAs, miRNAs, and mRNAs in EC through TCGA database. Subsequently, we constructed 111 lncRNAs (62 downregulated and 49 upregulated), 11 miRNAs (8 downregulated and 3 upregulated), and 63 mRNAs (33 downregulated and 30 upregulated) were included in the ceRNA network. Here, we are concerned about the relative regulation of lncRNAs in the ceRNA network. We have constructed the PPI network and found the key genes in the network. Our results indicated that KAT2B, EZH2, RUNX2, COL1A1, E2F3, CBFB, EGR2, GATA6, NFIC, and HMGA2 had important roles in the ceRNA network. These genes may play an important role in EC [37–40]. Here, we identified lncRNAs related to prognosis from related DElncRNAs in the ceRNA network. Seven lncRNAs are considered to be related to the prognosis of EC. HOTAIR has been confirmed to have higher expression levels in ESCC tissues than in the corresponding noncancerous tissues in these seven lncRNAs. Increased HOTAIR expression is related to poor prognosis. In the clinical cohort study, it was also found that HOTAIR has corresponding prognostic value in ESCC [13, 14]. DSCR8 has been shown to be involved in the progression of cancer. DSCR8 can activate the Wnt/*β*-catenin signaling pathway to promote HCC progression through the DSCR8/miR-485-5p/FZD7 axis [41]. Similarly, DSCR8 has been shown to be dysregulated in ovarian cancer [42]. In our study, the upregulation of DSCR8 changes significantly, which means that it may play an important role in the occurrence of EC. DIRC3 is upregulated in melanoma and may be used as an inhibitor of melanoma. But DIRC3 has not been shown to be significantly different in EC [43]. LINC00365 is upregulated in colorectal cancer specimens, and it may be involved in the process of CRC cells by mediating the Wnt/*β*-catenin pathway [44]. However, the expression of LINC00365 was downregulated in our study, which may be caused by different cancer types. The other three lncRNAs have not been reported; they may be newly discovered lncRNAs.

Subsequently, we constructed a prognostic model of EC through univariate and multivariate cox regression models. Three lncRNAs were included in the model. The prognostic model constructed by these three lncRNAs showed good predictive ability.

## 5. Conclusions

Our research is based on TCGA database to screen differentially expressed lncRNAs, miRNAs, and mRNAs in esophageal cancer. Then, the ceRNA network containing lncRNAs was constructed, which was used to find seven lncRNAs that related to the prognosis of esophageal cancer. Finally, a prognostic model of esophageal cancer containing three lncRNAs was constructed. These lncRNAs may be used as prognostic markers of esophageal cancer. In conclusion, our research will provide new insights into the regulation of lncRNAs in esophageal cancer.

## Figures and Tables

**Table 1:** DElncRNAs, DEmiRNAs, and DEmRNAs included in the ceRNA network.

Category	Changes	Gene symbol
lncRNAs	Downregulated	TTTY14, C9orf106, LINC00304, WDFY3-AS2, SPATA8, C5orf60, AL162511.1, AC005082.1, LINC00269, AP000897.1, AC079467.1, SNHG14, POU6F2-AS1, LINC00365, THRB-IT1, LINC00457, LINC00113, PCA3, PRICKLE2-AS3, DNMBP-AS1, PCDH9-AS2, TTTY10, ZRANB2-AS2, C9orf147, DIRC3, F10-AS1, MIR497HG, CYP1B1-AS1, TTLL7-IT1, LINC00472, ENOX1-AS1, JAZF1-AS1, MAGI2-AS3, ZRANB2-AS1, SRGAP3-AS4, MACROD2-AS1, DAPK1-IT1,CADM2-AS1, ZBTB20-AS3, MAGI1-AS1, ADAMTS9-AS1, ADAMTS9-AS2, AL353803.1, AC114810.1, AC021755.3, AC018926.1, AP001094.1, AC007389.1, FOXP1-AS1, AL391807.1, AP005717.1, AC012181.1, LIFR-AS1, ALDH1L1-AS2, FAM13A-AS1, DNAH10OS, C8orf49, AC110619.1, RMST, AC135776.1, PWRN1, LINC00261
	Upregulated	PVT1, DLEU2, SNHG1, LINC00460, GK-IT1, ALMS1-IT1, LINC00337, SNHG15, ZEB1-AS1, SNHG3, POU6F2-AS2, AL391152.1, AC007611.1, C17orf82, LINC00184, HOTAIR, LINC00392, C15orf54, AP002478.1, C8orf31, TM4SF19-AS1, HCP5, AC009093.1, AL513123.1, DLX6-AS1, FNDC1-IT1, LINC00393, AC131254.1, LINC00355, AL118508.1, AC131157.1, CASK-AS1, LMO7-AS1, AC093515.1, AC019294.2, HOTAIRM1, AC004917.1, NAALADL2-AS2, AC123768.1, MYO16-AS1, LINC00299, LINC00491, LINC00114, DSCR8, LSAMP-AS1, LINC00237, E2F3-IT1, LGALS8-AS1, KTN1-AS1
miRNAs	Downregulated	hsa-miR-139-5p, hsa-miR-338-3p, hsa-miR-125a-5p, hsa-miR-125b-5p, hsa-miR-129-5p, hsa-miR-490-3p, hsa-miR-363-3p, hsa-miR-135a-5p
	Upregulated	hsa-miR-17-5p, hsa-miR-301b-3p, hsa-miR-508-3p
mRNAs	Downregulated	NR3C2, VLDLR, TXNIP, CADM2, ADAMTSL3, KAT2B,RBM47, RBPMS2, GRM7, DBT, SCRG1, PTF1A, NFIC, CNTN4, DCLK2, ZNF385B, GLUL, LPP, SATB1, TTC28, THRB, NTN4, RGMB, CRY2, STARD13, GAB2, LIFR, FAM174B, LIN28A, BTG2, GATA6, NOVA1, PDE4D
	Upregulated	MMP11, EZH2, CBFB, BCL2L12, E2F3, DUSP10, HAUS8, CENPQ, COL1A1, ZNF367, ADAM17, CEP19, RUNX2, TNFAIP3, HOXD10, SEMA7A, ASPN, FJX1, DUSP6, HMGA2, COL5A1, ARID3A, CAMK2N2, EIF4EBP1, MSN, STON2, EGR2, HOXA10, ETV1, NEUROD2

**Table 2:** MCC method to calculate the key genes and their scores in the PPI network.

Rank	Gene symbol	Score
1	KAT2B	14
2	EZH2	12
3	RUNX2	9
4	COL1A1	4
5	E2F3	4
6	CBFB	4
7	EGR2	4
8	GATA6	4

## Data Availability

The Cancer Genome Atlas (TCGA) database (https://cancergenome.nih.gov/) is used to provide RNA-seq data, microRNA data, and the clinical data of EC.

## Supplementary Materials

Supplementary Materials Supplementary File 1: the R code and the corresponding running results to build the multiple COX regression model.
