# Supplementary material for: Comprehensive Analysis of lncRNAs Related to the Prognosis of Esophageal Cancer Based on ceRNA Network and Cox Regression Model
Source: Biomed Res Int. 2020 Dec 10;2020:3075729. doi: 10.1155/2020/3075729 (PMC7748909; doi:10.1155/2020/3075729)

**R code:**

library(survival)

dir="C:\\Users\\lixiaochao\\Desktop\\5. multiCoxinput "

setwd(dir)

inputfile="multiCoxinput.txt"

lncRNA<-read.table(inputfile,header=T,sep="\t",row.names = 1,check.names = F,stringsAsFactors = F)

lncRNAEXP=log2(lncRNA[,3:ncol(lncRNA)]+1)

lncRNA=cbind(lncRNA[,1:2],lncRNAEXP)

lncRNA[,"survival_time"]=lncRNA[,"survival_time"]/365

fmla1 <- as.formula(Surv(survival_time,status)~.)

mycox <- coxph(fmla1,data=lncRNA)

mycox<-step(mycox,direction="both")

risk_score<-predict(mycox,type="risk",newdata=lncRNA)

risk_level<-as.factor(ifelse(risk_score>median(risk_score),"High","Low"))

write.table(cbind(id=rownames(cbind(lncRNA[,1:2],risk_score,risk_level)),cbind(lncRNA[,1:2],risk_score,risk_level)),"risk_score.txt",sep="\t",quote=F,row.names=F)

summary(mycox)

install.packages("survminer")

library(survminer)

pdf("forest1.pdf",12,8)

ggforest(mycox,fontsize = 1)

dev.off()

**Code running result:**


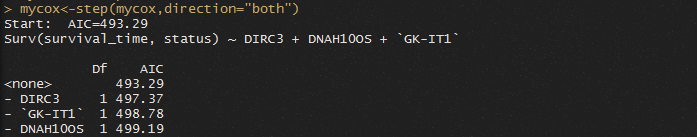


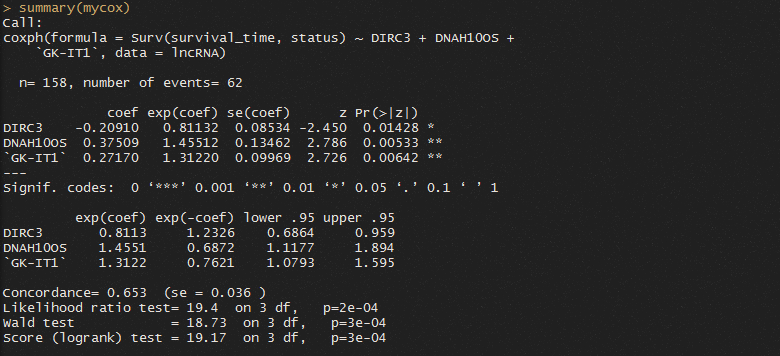

Supplement: Supplementary Materials — Supplementary File 1: the R code and the corresponding running results to build the multiple COX regression model. [file 3075729.f1.docx]
